# The Genomic Landscape of Prostate Cancer

**DOI:** 10.3390/ijms140610822

**Published:** 2013-05-24

**Authors:** Lien Spans, Liesbeth Clinckemalie, Christine Helsen, Dirk Vanderschueren, Steven Boonen, Evelyne Lerut, Steven Joniau, Frank Claessens

**Affiliations:** 1 Molecular Endocrinology Laboratory, Department of Cellular and Molecular Medicine, University of Leuven, Campus Gasthuisberg, Herestraat 49, P.O. Box 901, 3000 Leuven, Belgium; E-Mails: lien.spans@med.kuleuven.be (L.S.); liesbeth.clinckemalie@med.kuleuven.be (L.C.); christine.helsen@med.kuleuven.be (C.H.); 2 Division of Clinical and Experimental Endocrinology, Department of Clinical and Experimental Medicine, University of Leuven, Campus Gasthuisberg, Herestraat 49, P.O. Box 902, 3000 Leuven, Belgium; E-Mail: dirk.vanderschueren@med.kuleuven.be; 3 Division of Gerontology and Geriatrics, Department of Clinical and Experimental Medicine, University of Leuven, Campus Gasthuisberg, Herestraat 49, P.O. Box 7003, 3000 Leuven, Belgium; E-Mail: steven.boonen@med.kuleuven.be; 4 Division of Translational Cell and Tissue Research, Department of Imaging and Pathology, University of Leuven, Minderbroedersstraat 12 blok q, P.O. Box 1032, 3000 Leuven, Belgium; E-Mail: evelyne.lerut@med.kuleuven.be; 5 Division of Screening, Diagnostics and Biomarkers, Department of Development and Regeneration, University Hospitals Leuven, Campus Gasthuisberg, Herestraat 49, P.O. Box 7003, 3000 Leuven, Belgium; E-Mail: steven.joniau@uz.kuleuven.be

**Keywords:** prostate cancer, next-generation sequencing, copy number changes, gene fusions, long non-coding RNAs, methylation, microRNAs, single nucleotide polymorphisms, single nucleotide variants

## Abstract

By the age of 80, approximately 80% of men will manifest some cancerous cells within their prostate, indicating that prostate cancer constitutes a major health burden. While this disease is clinically insignificant in most men, it can become lethal in others. The most challenging task for clinicians is developing a patient-tailored treatment in the knowledge that this disease is highly heterogeneous and that relatively little adequate prognostic tools are available to distinguish aggressive from indolent disease. Next-generation sequencing allows a description of the cancer at an unprecedented level of detail and at different levels, going from whole genome or exome sequencing to transcriptome analysis and methylation-specific immunoprecipitation, followed by sequencing. Integration of all these data is leading to a better understanding of the initiation, progression and metastatic processes of prostate cancer. Ultimately, these insights will result in a better and more personalized treatment of patients suffering from prostate cancer. The present review summarizes current knowledge on copy number changes, gene fusions, single nucleotide mutations and polymorphisms, methylation, microRNAs and long non-coding RNAs obtained from high-throughput studies.

## 1. Introduction

Prostate cancer (PCa) is the most common non-skin malignancy in men, with an estimate of 900,000 men diagnosed worldwide with PCa in 2008. It is the sixth most common cause of cancer-related mortality in men worldwide, estimated to be responsible for 258,000 deaths in 2008 [1]. Serum levels of prostate-specific antigen (PSA) have been used as a first diagnostic tool. PSA is a glycoprotein that is secreted by prostate epithelial cells, meaning that PSA values can be elevated both in benign and malignant conditions of the prostate. Additionally, PSA levels are not elevated in all cases of PCa. Although the PSA test was the major screening tool for PCa detection, it has drawbacks, as 30%–50% of patients are being overtreated and instead could be followed by active surveillance, while other patients are being undertreated [2]. Clearly, alternative diagnostics are needed.

Nowadays, urologists use pre-treatment risk stratification models, like the Partin tables, d’Amico risk groups and Kattan nomograms, which combine serum PSA level, clinical staging and biopsy Gleason score in order to better predict pathological stage at radical prostatectomy and the risk of disease recurrence following definitive local treatment [3–5]. Unfortunately, these stratification models have accuracies of only 75%–85% and do not take into account the heterogeneity in genetic, molecular and physiological characteristics of the disease. Indeed, the reasons why some cancers progress slowly, while others behave more aggressively, are not well understood. At present, we do not have markers to discriminate both types. Further improvement of the clinical management of PCa will only be possible by identifying biomarkers of PCa aggressiveness, which will not only enable a better selection of patients who will benefit from radical treatment, but will also reduce the overtreatment of patients with indolent PCa. The development of such patient-tailored treatments into daily clinical practice first requires thorough molecular characterization of the tumors and their biology.

From a molecular point of view, cancer can result from a combination of single nucleotide variants (SNVs), small insertions and deletions (indels), rearrangements, aberrant methylation and changes in copy number, which thus lead to differences in expression of oncogenes or tumor suppressor genes. Recent advances in massive parallel sequencing technologies allow for the detection of all of the aforementioned changes, at a much greater sensitivity and, importantly, also, at a constantly decreasing cost. To increase the speed of analyses, one can choose exome sequencing which allows the identification of SNVs and indels that affect the encoded proteins. Sequencing the whole genome, however, allows the additional detection of rearrangements and copy number changes. Alternatively, transcriptome sequencing not only provides data on gene expression, but can also be used to detect gene rearrangements at a lower cost than genome sequencing. High-throughput sequencing of immunoprecipitated methylated tumor DNA can identify aberrant methylation. All these techniques generate vast amounts of data that now need to be searched for correlations with disease outcome or responsiveness to specific treatments. In the long run, obtaining an entire (epi)genomic and transcriptomic landscape of PCa should assist in a better selection of therapy tools and can even contribute to the identification of targets for novel anticancer drug development.

## 2. Copy Number and Gene Expression Changes

The study of biomolecules coming from different sources is difficult, because tissues have undergone different preservation protocols [6]. DNA is, however, highly stable, which enables the detection of genome-wide copy number alterations (CNAs), genome-wide SNP analyses, whole exome sequencing or even whole genome sequencing [7]. CNAs can result in the amplification of oncogenes or the deletion of tumor suppressors, and these changes could contribute significantly to cancer etiology. Global analyses of copy number profiles of primary tumors and metastases identified recurrent aberrations associated with PCa development and progression, including broad losses of 1p, 6q, 8p and 9p and losses of large regions of chromosomes 13, 15, 18 and 22 [8]. Gains of 1q, 3q, 7q and 8q are also well described in PCa [8]. In addition, focal amplifications of the androgen receptor (AR) (Xq12) and homozygous focal deletions of PTEN (10q) and NKX3.1 (8p) are also frequent in PCa [7,9–11]. A recent more comprehensive CNA study of 218 primary and metastatic tumors by Tailor *et al.* confirmed the earlier data, but added a significant role for somatic copy number increases of the NCOA2 gene, which encodes an AR coactivator (see also Section 4.1.1) [10]. Similarly, copy number variations of CHD1 occur in 8% of lethal castration-resistant PCa (CRPC) samples [11]. CHD1 encodes an ATP-dependent chromatin-remodeling enzyme, previously reported as deregulated in PCa [12].

PCa is a clinically heterogeneous disease, meaning that the majority of cancer-affected prostates harbor multiple distinct primary tumor foci with different characteristics. High-resolution copy number changes from both primary tumor and different metastases revealed identical copy number changes, shared by all same-case cancer foci and defined by the same breakpoints in all multi-tumor cases [13]. This suggests that the genome copy number architecture was extremely homogeneous and conserved both within the primary tumor and between primary and metastatic tumors [14]. This also indicates that metastatic PCas have monoclonal origins and maintain the unique signature copy number pattern of the parent cancer clone [13,15]. However, each focus will also accumulate a variable number of separate subclonally sustained genomic changes. So, although multiple tumor foci commonly arise from a single clone, this does not imply that the separate foci are biologically homogeneous. In conclusion, it is to be expected that multiple primary foci within one prostate indeed have the same genetic origin, although they may, to some extent, acquire distinct genetic lesions.

Another study reported an increasing percentage of the genome affected by CNAs with increasing stage, grade and diagnostic PSA levels [16]. This is in agreement with the study from Taylor and colleagues, who reported that metastases harbor more whole chromosome, chromosome arm and focal amplifications and deletions than primary tumors [10]. The distinct subclass of tumors with ERG rearrangements (described in the next section) was associated with 7q gain and 16q deletion, while 6q deletion was enriched in non-rearranged cases [9]. This 6q loss in non-rearranged PCa is accompanied by deregulation of the MYO6 gene [9]. Another study revealed three regions of recurrent copy number loss associated with the TMPRSS2-ERG fusion: two regions spanning the tumor suppressors PTEN and TP53, respectively and a third spanning the multigenic region at 3p14 [10]. These data revealed distinct subgroups with substantial differences in time to biochemical (PSA) relapse. More specifically, two subgroups of primary tumors were defined, those with minimal CNAs and those with substantial alterations. The latter group included most of the metastatic samples with unfavorable prognosis [10]. Importantly, there is no correlation between high Gleason scores and these two subgroups, indicating that histology and copy number alterations are non-overlapping features [10]. Hence, CNA could become useful as an additional clinical marker independent from Gleason scores.

## 3. Gene Fusions

A second type of molecular alterations occurring in cancer is the fusion or rearrangement of genes. A large number of chromosomal rearrangements were primarily discovered in leukemias, lymphomas and sarcomas [17]. The first report on gene rearrangements in solid tumors in general and PCa in particular, however, was reported in 2005, when Tomlins and colleagues applied a statistical approach termed cancer outlier profile analysis in combination with rapid amplification of cDNA ends, thus identifying the TMPRSS2-ERG, TMPRSS2-ETV1 and TMPRSS2-ETV4 fusions in PCa samples [18,19].

### 3.1. Detection of ETS Gene Fusions in PCa

The ERG, ETV1 and ETV4 genes belong to the family of v-ets erythroblastosis virus E26 oncogenes (ETS), which encode transcription factors characterized by a highly conserved, sequence-specific DNA-binding domain, the so-called ETS domain [20]. The TMPRSS2 gene encodes an androgen-regulated, type II transmembrane-bound serine protease that is highly expressed in normal prostate tissue, as well as in neoplastic prostate epithelium [21,22]. This explains why the gene fusion leads to the androgen-responsive, prostate-specific expression of these ETS transcription factors. The recurrent TMPRSS2-ETS fusion is by far the most common rearrangement described in any neoplasm, since it has been found in approximately 50% of all PCa cases examined [23]. In terms of morphological features, blue-tinged mucin, a cribriform growth pattern, macronucleoli, intraductal tumor spread and signet-ring cell features are associated with the occurrence of the TMPRSS2-ERG fusion [24].

Less common genomic rearrangements in PCa were identified later and involved SLC45A3, HERV-K, HNRPA2B1, KLK2 and C15orf21 as 5′ fusion partners of ETV1 and FKBP5 as a fusion partner of ERG [25,26]. SLC45A3 is a prostate-specific androgen-responsive gene that has been found fused to ERG, ETV1, ETV5 and ELK4 [25,27–31]. Recently, a SNURF-ETV1 fusion formed in conjunction with a complex rearrangement event was detected. It involves the androgen-regulated 5′ fusion partner, SNURF, and it also led to marked overexpression of ETV1 [32]. An overview of all ETS gene fusions identified so far in PCa samples can be found in Table 1.

In general, the ETS transcription factors are considered poor therapeutic targets owing to their lack of enzymatic activity, their inaccessibility because of intranuclear activity and their dependence on interactions with other proteins to achieve specificity. Nevertheless, attempts are being made to develop compounds that interfere specifically with the function of ETS genes as transcription factors [40]. Alternatively, inhibitory molecules that target the TMPRSS2 promoter and/or control regions could also reduce ETS expression.

### 3.2. Detection of Non-ETS Gene Fusions in PCa

Paired-end transcriptome sequencing identified several other rearrangements involving genes of the RAF kinase pathway: SLC45A3-BRAF, AGTRAP-BRAF, ESRP1-RAF1, EPB41-BRAF and RAF1-ESRP1 [41,42]. Some of the proteins encoded by these gene fusions, like BRAF, are well known drug targets, so the expression of these genes might become clinically useful in the future. Many other non-ETS gene fusions have been identified, although each of these fusions was detected only once [14,26,27,32,39]. Moreover, two novel 3′ fusion partners of TMPRSS2 have been identified: FKBP5 and CCDC21 [26,32]. Validation of the fusions involving FKBP5 led to the discovery of a complex triple fusion event with FKBP5 joined to TMPRSS2 and ERG [26]. In general, the non-ETS aberrations can occur both in TMPRSS2-ERG negative and positive cancers.

It is becoming increasingly clear that most of the genomic fragments that are fused to the protein-coding part of ETS genes cover transcription control regions that direct prostate-specificity and androgen-responsiveness. This has been shown for TMPRSS2, SLC45A3, EST14, HERVK17, HERPUD1, C15orf21, FLJ35294, NDRG1, ACSL3, FKBP5, KLK2 and CANT1. However, some 5′ partners of ETS-fusions, like DDX5 and C15orf21, are ubiquitously expressed and androgen-insensitive, indicating that non tissue-specific promoter elements can also drive ETS gene overexpression [25,29]. Adversely, most non-ETS gene fusion partners are not androgen-regulated, unless they are part of a complex fusion event with ETS fusions.

### 3.3. The Role of Fusion Genes in the Molecular Pathology of PCa

Although there are more non-ETS gene fusions identified than ETS gene fusions, most of these have been detected only once. This is in contrast to the ETS gene fusions, which occur at high frequencies in PCa patients, ranging from 15% to 70%, depending on the clinical cohorts investigated. Of these ETS fusions, ERG rearrangements were identified in 53% of 540 patients [28]. After ERG, ETV1 is the most commonly rearranged in about 5% of the patients [35]. Other ETS genes, such as ETV4 and ETV5, may have rearrangement frequencies at or below 1%–2%.

The impact of the occurrence of fusions on prognosis has been investigated in many clinical studies, but remains highly debated. A recent study involving 1039 radical prostatectomy tumors discovered that positive ERG rearrangement status is associated with younger age at diagnosis, lower serum PSA and lower prostate volume [43]. In another cohort of 2800 PCas, no relation was found between the ERG gene rearrangement and the clinical outcome or tumor phenotype [44]. Important to note is the Edel subclass in which the TMPRSS2-ERG fusion is generated by interstitial deletion. This fusion type has been correlated with aggressive PCa and poor prognosis in two separate cohorts of 30 and 445 patients, respectively [45,46].

Next-generation sequencing analyses of one aggressive primary tumor revealed seven novel fusion genes. The genes involved in these fusion events fell into two categories: one category with androgen-responsive (AR)-regulatory genes normally expressed in luminal cells and one with genes that are normally expressed in neuroendocrine cells [14]. This hybrid phenotype was apparent for the primary, as well as the metastatic tumors of this patient. Wu and colleagues, thus, may have discovered a novel type of highly aggressive PCa and suggested that chromosomal translocations are cell-type specific, as they occur preferentially in transcriptionally active genes [12,14,39].

While the existing paradigm dictates that chromosomal rearrangements occur gradually over time, recent evidence suggests that in some cancers tens to hundreds of genomic rearrangements involving only one or a few chromosomes can occur in a cellular crisis resulting in cancer-causing lesions. This phenomenon, known as chromothripsis, was first described by Stephens and colleagues in a patient with chronic lymphocytic leukemia and several cancer cell lines [47]. In PCa, chromothripsis was reported one year later and was detected by the presence of triple fusion genes [39]. Whole genome sequencing of seven high risk primary tumors revealed complex inter- and intra-chromosomal events involving an exchange of “breakpoint arms” generating a mix of chimeric chromosomes. There was, however, no loss of genetic material in contrast to what happens during chromothripsis [12]. These complex translocations will deregulate multiple genes in parallel, and this may drive prostate tumorigenesis. Very recently, these rearrangement chains have been termed chromoplexy [48].

The homogeneous distribution of the TMPRSS2-ERG fusion and its presence in 19% of high-grade prostatic intraepithelial neoplasia (PIN) lesions adjacent to cancer foci suggests that this fusion is an early event in the development of invasive PCa [23]. In some TMPRSS2-ERG-positive tumors, rearrangement breakpoints occur preferentially within regions containing AR and ERG DNA binding sites, while in ETS fusion-negative cells, there is an inverse correlation with these regions, indicating alternative mechanisms for the genesis of breakpoints. This suggests a causal link between the open chromatin structure linked to transcriptional activities at the genes involved and the mechanism of translocation [12]. A recent study performed whole genome sequencing on 11 patients with early onset PCa [32]. Despite an overall lower number of structural rearrangements in early onset PCa compared to elderly onset PCa, they detected an increase in balanced rearrangements and a higher fraction of gene rearrangements also affecting androgen-driven genes in early onset PCa [32]. This contrasts with the accumulation of non-androgen-associated structural rearrangements in elderly onset PCa, most of which correspond to copy number alterations with concomitant loss of genetic material. In terms of consequences for the oncogenic process, the authors conclude that most early onset PCas involve an androgen-driven pathogenic mechanism characterized by a marked abundance of balanced DNA structural alterations involving androgen-regulated genes [32].

The role of ERG overexpression in PCa development has been studied in transgenic mice expressing the ERG gene fusion product under androgen-regulation. These mice only develop PIN-like structures [49]. Thus it seems clear that the TMPRSS2-ERG fusion on its own is insufficient to induce the development of invasive carcinoma, indicating that other (epi)genetic factors also contribute to the initiation of PCa. However, the presence of the gene fusion between TMPRSS2 and ERG promotes PCa in both mouse and humans when PTEN is concurrently lost [50–52]. Similarly, a feedback control with the AR pathway has been described: while ERG expression from the TMPRSS2-ERG fusion is androgen-induced, ERG itself shuts down androgen signaling, inhibits normal prostate differentiation and turns on EZH2 expression. The latter in turn induces an embryonic stem cell-like dedifferentiation program, which might initiate tumorigenesis [53].

In conclusion, although a lot is known about the gene fusions in PCa, from a clinical point of view, further classification tools, probably independent, as well as dependent of the fusion status, are needed to help determine the optimal patient-tailored treatment modalities. For some cases, like BRAF-fusion positive PCa, an optimal treatment with BRAF kinase inhibitors is already available in the clinic.

## 4. Single Base Pair Changes

### 4.1. Single Nucleotide Variants (SNVs)

#### 4.1.1. The Beginning of Next-Generation Sequencing

Targeted resequencing of 157 genes in 80 primary tumors and metastases confirmed that the AR was the most frequently mutated gene in PCa metastases [10]. While it is known that alteration of the AR through mutation, gene amplification or overexpression occurs exclusively in metastatic samples after hormone therapy, alterations of the AR pathway also occur in 56% of high volume primary tumors and were confirmed in 100% of the metastases [54]. In addition, the nuclear receptor coactivator NCOA2 had a gain of expression or mutation in 8% of primary tumors and 37% of metastases. Integration of all the mutation data with copy number alterations and transcriptome data revealed that three well-known cancer pathways were commonly altered: PI3K, RAS/RAF and RB [10].

The resequencing of 577 genes implicated in cancer in eight metastases from six patients identified 14 coding mutations. Again, the characteristics of the genes that underwent copy number variation or mutation supported a major role for the AR pathway in PCa for at least half of the cases [55].

To study genomic changes in PCa, one can also study cell lines or tumors either grown *in vitro* or as xenografts in immunocompromised mice. This has the advantage that the response to cancer-directed therapeutics can be monitored, but the disadvantages are that no corresponding normal tissue or DNA is available and that a number of the genomic changes will have arisen during the culturing that were not present in the original metastatic tissues [56]. Despite this, TP53 was the most frequently mutated gene in the xenografts and pathway analysis of genes mutated in castration-resistant compared to castration-sensitive pairs of tumor lines derived from the same PCa revealed a significant enrichment of the Wnt signaling pathway [56]. Exome sequencing of the LNCaP PCa cell line revealed 1,802 non-synonymous SNVs, while a median of only 30 SNVs is detected in the exome of primary PCas [57,58]. The difference in the amount of detected SNVs can be attributed to DNA mismatch repair deficiency in LNCaP cells, on the one hand, and the acquisition of genomic changes during culturing, on the other hand [59,60].

#### 4.1.2. Large Scale Genomic Analyses

**Figure 1:**
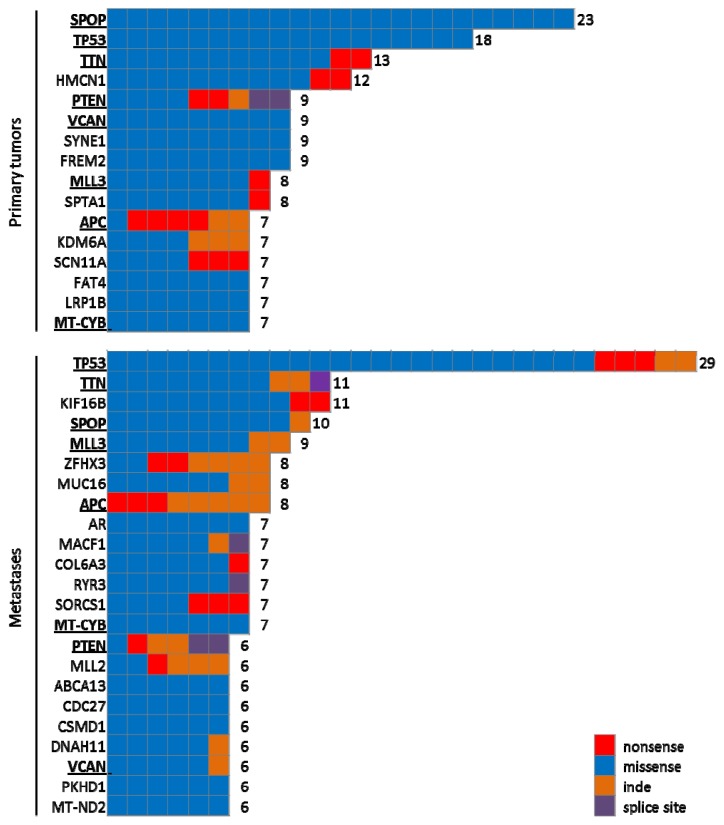
Overview of the most frequently mutated genes in primary and metastatic prostate cancer. Gene lists were taken from [10–12,32,42,55,58,69,70]; the cumulative number of mutations is given on the right. The names of genes that are recurrently mutated both in primary tumors and in metastases are bold and underlined.

Berger and colleagues reported an average of 20 non-synonymous SNVs in seven high-risk primary PCas [12]. Only the SPTA1 gene, involved in erythroid cell shape specification, was mutated in two out of seven tumors [12]. More recently, two studies explored the presence of SNVs in 112 primary tumors and 50 metastases, respectively. Both studies performed whole exome sequencing and reached an average of 120-fold coverage [11,58]. Here, a median of 30 non-synonymous SNVs in the exome of primary PCas was detected. Most likely, the higher exome sequence coverage in the latter two studies improved the detection of SNVs that are present at lower allelic fractions and, thus, explains the higher number of SNVs [58]. Indeed, a recent genomic study with 30–40x coverage on 11 samples detected an average of only 16 non-synonymous SNVs (ranging from three to 55) [32]. Barbieri and colleagues reported twelve genes, which were recurrently mutated and contained more mutations in PCa than expected by chance: PIK3CA, PTEN, TP53, SPOP, FOXA1, MED12, CDKN1B, ZNF595, THSD7B, NIPA2, C14orf49 and SCN11A (Figure 1) [58]. The PIK3CA, PTEN and TP53 genes were already well known to be involved in the tumorigenesis of PCa, but several genes not previously known to undergo somatic alteration in PCa were enriched for mutations, including FOXA1, MED12, THSD7B, SCN11A, NIPA2, C14orf49 and ZNF595 [58]. Some of these genes affect the androgen signaling axis. The transcription factor FOXA1 regulates cell proliferation and promotes tumor progression in CRPC [61]. Moreover, it can act as a pioneering factor for AR binding to chromatin, and the protein level in primary tumors has been associated with disease outcome [62]. Mutations affecting MED12 were not previously observed in PCa, but had been reported in 70% of uterine leiomyomas [63]. MED12 is a subunit of the mediator complex that regulates transcription by bridging DNA regulatory sequences to the RNA polymerase II initiation complex [64]. CDKN1B was known to constrain prostate tumor growth in mice by inhibiting cell proliferation and cancer progression, but somatic substitutions had not been previously observed in this cell cycle regulator [65].

The SPOP gene was mutated in 13% of the analyzed tumors [58]. Also, in additional cohorts, an estimate of 6%–15% of the tumors contained a SPOP mutation. A novel study using Sanger sequencing detected the SPOP mutations in only 2% of PCa tumors [42], while we detected a mutation in four out of 75 primary tumors (unpublished data). In contrast to SPOP mutations detected in other cancers, which are scattered over the entire length of the protein, the PCa mutations cluster in the substrate-binding cleft. Remarkably, the presence of a SPOP mutation was mutually exclusive with mutations in TP53, PTEN or the TMPRSS2-ERG rearrangement [58]. SPOP encodes the substrate-binding subunit of an E3 ubiquitin ligase and, hence, is a modulator of stability for specific substrate proteins. Interestingly, the p160 coactivators of nuclear receptors are substrates for SPOP [66]. In this way, these mutations could affect the AR axis.

Grasso *et al.* performed another exome sequencing study of metastatic biopsies of 50 lethal, heavily pre-treated CRPCs and identified nine genes that were significantly mutated [11]. Of these, six were already reported as recurrently mutated in PCa: TP53, AR, ZFHX3, RB1, PTEN and APC [11]. Three other genes were novel for PCa: MLL2, CDK12 and OR5L1. MLL2 is a histone methyltransferase that mediates H3K4 trimethylation, which is recurrently mutated in multiple cancers [67]. The cyclin-dependent kinase CDK12 protects cells from genomic instability through regulation of expression of DNA damage response genes [68]. As OR5L1 encodes an olfactory receptor, a role of this mutation in the oncogenic process is more difficult to envision. Grasso *et al.* also found that CHD1 is mutated or deleted in 8% of PCas. Using Oncomine, they detected focal deletions or mutations of CHD1 in 5.2% of 954 PCas, 96% of which were negative for the ETS-fusion. This integrated analysis identifies CHD1^−^/ETS^−^ as a novel PCa subtype [11]. Together, their data suggest that aberrations in AR and interacting proteins, including chromatin/histone remodelers, ETS genes and known AR coregulators, including FOXA1 are common in CRPC [11].

Integration of exome sequencing on primary PCa with RNA sequencing and copy number alteration revealed that the mutation rate in the mitochondrial genome was 55-times higher than that of the autosomes [69]. More specifically, the electron transport chain was mutated in almost half of the tumors. Several of these mitochondrial SNVs were not yet reported, although closely related genes have been reported to be mutated in other cancers [69]. For example, MLL3 was a novel gene detected to be mutated in PCa, while its close relative MLL2 has been previously reported in PCa. However, the biological and clinical relevance of these mutations needs to be documented further.

In conclusion, with over 200 PCa sequences reported, we can conclude that point mutations in PCa are not as rare as initially expected. While very few genes are recurrently affected, the mutations recur in specific signaling pathways, like the androgen signaling pathway. Moreover, until studies are undertaken in substantially larger cohorts, it will be difficult to attribute significance to the different SNVs. We therefore merged the lists of SNVs detected in the aforementioned studies, both for primary tumors and metastases. The most frequently mutated genes are represented in Figure 1.

#### 4.1.3. Future Perspectives

##### 4.1.3.1. The Use of FFPE Samples

All the aforementioned studies used DNA from fresh frozen specimens, while the majority of tissues available in pathology archives are formalin fixed paraffin embedded (FFPE). This formalin fixation induces cross-linking between cytosine nucleotides impacting the integrity of the DNA. Nevertheless, two studies used FFPE material to detect SNVs. A first study detected a drastic increase in transversion mutations: 4% in fresh frozen tissue compared to 30% in FFPE tissue from the same primary prostate tumor [71]. Despite this, the majority of the remaining SNVs were common in both samples. Targeted resequencing using as little as 55 nanogram of DNA from FFPE material detected more genomic alterations in CRPC compared to localized tumors. Additionally, a novel variant encoding the amino-terminal transactivation domain of the AR was detected [42].

In the near future, we hope that the use of FFPE samples becomes widespread, as this would open up the possibility to sequence material from thousands of patients for which detailed clinical data and long-term follow-up data are available for retrospective analyses.

##### 4.1.3.2. Prostate Cancer is a Multi-Focal Disease

Because of the clinical heterogeneity of PCa and the presence of multiple distinct primary tumor foci, it needs to be established whether these foci are identical, similar or arose independently. Also, it has to be established whether the so-called index lesion is indeed of clinical relevance and harbors the site where metastases evolve from. In one study, four primary tumors, three of which harbored multiple foci, were sampled to verify the monoclonal or polyclonal origin of these different foci. No common SNVs were detected in the different foci of these primary tumors, indicating that the foci are independent cancers [70]. Profiling of three foci from another patient identified overlapping CNA regions, but no identical breakpoints, suggesting that the CNAs identified in all three foci also were independent events [70]. Low coverage for variant calling and limited copy number analysis might be an explanation for these results that deviate from the more generally believed hypothesis that different foci have a common origin. This was also confirmed by other studies using copy number changes (discussed in Section 2).

For the development of correct DNA diagnostics, it will hence be necessary not only to study more matched primary-metastatic tumor pairs, but also to sample multiple separate tumor foci from within the same prostate. This should help to determine the molecular events that can occur during progression to advanced disease or, alternatively, may even help to identify less aggressive lesions.

### 4.2. Single Nucleotide Polymorphisms (SNPs)

The risk of developing PCa doubles for men with a first degree relative affected by PCa and increases further with more affected relatives [54]. This indicates that PCa is one of the most heritable cancers with up to 15% of cases linked to family history [72]. Twin studies similarly suggest that up to 42% of the risk for developing PCa is linked to heritable components, indicating that the contribution of genetic factors to the development of PCa is greater than to the development of other types of common human tumors [73].

Genome-wide association studies (GWAS) compare the frequency of common single nucleotide polymorphisms (SNPs) throughout the entire genome (minor allele frequency >1%–5% in the population) in PCa patients and controls. In a typical GWAS, up to one million SNPs are evaluated in large cohorts of thousands of patients *versus* controls to determine the link between specific forms of the SNPS and the probability to develop PCa. Because only 1 or 2 million of approximately 50 million SNPs are assessed, the SNPs associated with PCa through GWAS are unlikely to be the causal genetic risk variant. However, these risk-associated SNPs segregate with the underlying causal variant, since they are in linkage disequilibrium [74]. More than 70 PCa susceptibility loci explaining approximately 30% of the familial risk have been identified (see Table 2 for an overview) [6,75]. Additional case-control studies are generally needed to confirm the GWAS findings, as the risk of false positives is appreciable. The relative increased risk of developing the disease based on any single polymorphism discovered to date is small, generally <1.5-fold, but risk appears to increase with increasing number of risk alleles carried. A recent study evaluated 25,000 PCa cases and identified 23 novel PCa susceptibility loci [75]. Pathway enrichment of previously and newly reported susceptibility regions revealed overrepresentation in pathways regarding cell adhesion and extracellular matrix, transcriptional regulation by the AR and WNT, FGF and IGF signaling [75]. Prospective GWAS studies can also evaluate rarer variants (minor allele frequency ≤1%) associated with PCa risk, which may be more highly penetrant and carry higher relative risk.

The SNP risk markers can be located within protein-coding genes, in intergenic regions, in unannotated transcripts, such as lncRNAs or miRNAs, in regulatory regions or in loci without any known genes at all. There are several potential mechanisms by which these SNPs may be associated with altered PCa risk, including genetic linkage to a coding variant in a cancer-relevant gene, changes in promoter or enhancer binding sites, changes in chromatin structure that affects expression of adjacent or distant genes or changes in expression of noncoding RNAs [6]. Jin and colleagues demonstrated that eight of the known PCa-risk SNPs fall into the intervals of long noncoding RNAs [92].

The 8q24 region contains various independent PCa-susceptibility loci within a 1 Mb segment, and some of them were found to be significantly associated with other types of cancer, as well, including colorectal, breast, ovarian and bladder cancer. Surprisingly, no gene has been annotated in this 1 Mb region, and its biological significance in cancer remains unclear. A possible explanation is the presence of an enhancer, which physically interacts with the MYC oncogene in a tissue-specific manner [93]. Similarly, the 17q24 region harbors the rs1859962 SNP that is associated with PCa risk, and it defines a 130 kb linkage disequilibrium block that lies in a 2 Mb gene desert area [74]. This block contains the rs8072254 and rs1859961 functional SNPs, which modulate AR and AP-1 binding, respectively, leading to an increased transcriptional activity of the prostate-specific enhancer in this block that loops to the SOX9 oncogene [74].

Another SNP is located upstream of the MSMB gene. The PSP94 protein encoded by MSMB is found in semen, and its expression has been shown to be either lowered or lost in PCa. It could, therefore, be a biomarker for high risk PCa or progression [94,95]. Multiple SNPs in the promoter region of KLK3 have been associated with serum PSA levels, and some have been suggested to be associated with risk of PCa [96,97]. Very recently, a SNP in the intronic region of the TERT gene at 5p15 was identified that is associated with TERT expression [98]. These studies demonstrate the potential interaction between genetic variants and clinical outcome.

Subjects participating in most of the GWAS studies were recruited from the general population and, thus, primarily represent sporadic cancer cases. A study of SNPs in hereditary PCa indicated that at least a subset of PCa risk-related loci identified by case-control GWAS are also associated with disease risk in hereditary PCa [99]. Several GWAS studies revealed associations of rs11672691, rs6497287 and rs1571801 with more aggressive disease, which might make them useful as prognostic markers [100–102].

Although GWAS studies have revealed interesting aspects of PCa, the potential benefits of applying risk models based on SNPs in clinical practice are difficult. In the future, however, these genetic markers could be incorporated in clinical decision-making and take part in risk models, screening paradigms and treatment recommendations.

## 5. DNA Methylation

Epigenetics is the study of heritable changes in gene expression caused by mechanisms other than those inherited via the underlying DNA sequences. Here, we focus on DNA hyper- and hypo-methylation of cytosine-guanine (CpG) islands. DNA methylation can lead to gene-silencing either by inhibiting the access of target binding sites to the transcriptional activators or by promoting the binding of methyl-binding domain proteins, which interact with histone deacetylases that promote chromatin condensation into transcriptionally repressive conformations [103].

In general, overall DNA hypomethylation increases during tumor progression, while the specific hypermethylation of promoter regions of tumor suppressor genes is observed in both initiation and progression of PCa [104]. The best characterized gene of which the promoter is hypermethylated in more than 90% of PCas encodes the glutathione *S*-transferase P1 (GSTP1) gene. More than 60 genes have been reported to be differentially hypermethylated in progressive PCa [103,105]. Again, some of these genes have been shown to be involved in the androgen signaling pathway. Recently, the tumor suppressor miR-124 targeting the AR has been shown to be silenced by methylation in clinical PCa samples [106]. An example of a hypomethylated gene is the plasminogen activator urokinase gene. Its increased expression is associated with higher invasive capacity of PCa cells *in vitro* and increased tumorigenesis *in vivo* [103].

Earlier epigenetic studies focused on individual or small numbers of genes. The advent of next-generation sequencing now allows profiling of methylomes, defined as the total of all DNA methylations in the whole genome. For PCa, such genome-wide studies revealed hypermethylation of homeobox or T-box genes, the EFEMP1, FLT4, AOX1 and WFDC2 gene, and dysregulation of genes involved in TNF-α-dependent apoptosis [107–112]. Similar to the CNA conservation, the unique DNA methylation signature of the tumor/metastasis-initiating focus was shown to be closely maintained during metastatic dissemination [113]. The alterations in DNA methylation patterns that are associated with phenotypic changes in gene expression have a surprising strong tendency to be maintained within metastases in an individual patient [113].

Methylome analysis of 51 primary PCas identified 147,000 cancer-associated epigenetic alterations, of which 58% were hyper- and 42% hypo-methylated [114]. Tumors without the TMPRSS2-ERG fusion contain more differentially methylated regions than fusion-positive tumors, suggesting a more pronounced role for epigenetic mechanisms in fusion-negative tumors [114]. Similarly, tumors with ERG promoter DNA methylation had a significantly higher number of methylated genes than tumors that lacked ERG promoter DNA methylation, although there was no association between ERG promoter methylation and the presence of the TMPRSS2-ERG fusion [115].

Clearly, in combination with DNA mutations and gene fusions, DNA methylation markers hold great promise as a clinically useful diagnostic or prognostic parameter. One major open question is whether specific subtypes of the disease might be identified by combinations of hyper- and hypo-methylation events. This is one of the topics that might be answered by more comprehensive, genome-wide studies.

## 6. Non-Coding RNAs

In this review on genomics, we will not discuss transcriptome studies, which aim to identify gene signatures that can assist in the classification of different types of PCa. Several such signatures have been proposed and are under investigation for their clinical use (for example [116,117]). More recently, transcriptome data have been integrated with genome analyses [32]. We will focus on the novel types of non-coding RNAs that have been discovered more recently thanks to the development of high-throughput sequencing. In the future, we hope that miRNA-signatures will become available and that these signatures can help to classify PCa, just as protein-coding gene signatures already can.

### 6.1. MicroRNAs

MicroRNAs are small, non-coding RNA molecules that bind to the 3′ untranslated region (UTR) of mRNA. This binding effectively silences translation by blocking access to the ribosome or by marking the target mRNA for degradation. Genes encoding miRNAs are found as independent entities or within introns of other genes, within repetitive genomic elements or within transposable element sequences.

At present, more than 100 miRNAs have been reported to be deregulated in PCa. There are, however, many conflicting results in the literature, which is likely due to the still immature technology to capture and quantitate miRNAs and the contamination of normal cells in the tumor samples [118–121]. Despite these inconsistencies, all studies confirm the widespread dysregulation of miRNAs in PCa. Moreover, a subset of these have been experimentally shown to be involved in the initiation, progression from androgen dependent to androgen independent stage, invasion and/or metastasis of PCa (reviewed in [122]). A growing number of miRNAs is being identified as interfering with the AR pathway. A gain-of-function screen in PCa cell lines identified 71 unique miRNAs that influence the level of AR in these cells, with 13 miRNAs validated in 3′ UTR-binding assays [123]. On the other hand, androgens control the upregulation of miR-125, miR-21 and miR-141 and, consequently, the downregulation of their respective target mRNAs [124–126].

Oncomirs are miRNAs that are dysregulated in cancer. Examples of miRNAs that show decreased expression in PCa compared to normal prostate tissue are miRNA-143, -145 and -200. The mRNA targets for these miRNAs are being discovered, and it seems that the miRNA downregulation results in epithelial-to-mesenchymal transition (EMT). More specifically, the miR-200 family is found to regulate EMT by targeting the E-cadherin repressors ZEB1 and ZEB2 [127–129]. Also, miRNA-143 and miRNA-145 inhibit tumor cell invasion and migration [130–132]. Conversely, miRNAs that show increased expression are miRNA-21 and miRNA-125b as they play important roles in resistance to apoptosis [133,134]. Here too, although some downstream mRNA targets are being discovered, most of them remain poorly understood. So far, many studies examining the role of miRNAs are associative and rely on PCa cell lines as surrogates for clinical response. It is thus envisaged that the focus will shift towards clinically relevant studies both in animals and humans to provide a better understanding of the working mechanisms of miRNAs [122]. Alternatively, studies in humans could pinpoint those microRNAs that can be used as a prognostic marker. In this way, miR-221 was discovered to be progressively downregulated in primary PCa and metastasis [135]. This downregulation is associated with Gleason score, tumor progression and clinical recurrence during follow-up [135].

Interestingly, certain microRNAs are not only elevated in the prostate tumor, but also in the circulation (for example in the exosomes) of the patient, suggesting they act similar to hormones and might play a role in priming the site of metastasis. An example is the upregulation of miR-375, which can predict biochemical relapse, with high expression being associated with an unfavorable outcome [136]. Circulating miRNAs open up the possibility of their development into diagnostic tools. Ultimately, miRNAs could be used to predict outcome and response to treatment or even be targets of treatment themselves.

### 6.2. Long Non-Coding RNAs (lncRNAs)

LncRNAs share common traits with mRNA, because they are mostly transcribed by RNA polymerase II, they are capped, polyadenylated and spliced, but they do not contain an open reading frame. Most of the lncRNAs identified to date display overexpression in PCa samples. A few lncRNAs seem to be prostate-specific: PCA3/DD3, PCGEM1, PRNCR1 and PlncRNA-1.

The best documented is PCA3, which was discovered as a differentially expressed RNA (DD3). It is expressed exclusively in the prostate, is highly overexpressed in PCa and detectable in urine samples of PCa patients [137]. PCA3 has been conclusively shown to be a better biomarker for PCa in biopsy samples than PSA. PCA3 and PSA together are an even better predictor of PCa [138]. This led to the FDA approval of a PCA3 urine test as diagnostic aid to decide on repeated biopsy testing.

Other lncRNAs are still in the early discovery phase. PCGEM1 also encodes an androgen-regulated lncRNA that is expressed exclusively in glandular epithelial cells of both normal and tumor specimens of human prostate [139]. In patients, tumor-associated overexpression of PCGEM1 was detected in 84% of the samples [139]. Probably the least characterized lncRNA is PRNCR1 (prostate cancer non-coding RNA 1), an approximately 13 kb intron-less non-coding RNA transcribed from the 8q24 region [140]. In a small cohort, the PRNCR1 expression was up-regulated in half of the PCa samples, as well as in the precursor PIN lesion. Finally, PlncRNA-1 was found to be overexpressed in 11 out of 16 PCa samples, and a knockdown resulted in decreased cell viability, increased apoptosis and a decrease of AR mRNA and protein [141].

Transcriptome analyses of a cohort of 81 prostate tissues led to the discovery of 121 unannotated PCa-associated lncRNA transcripts (PCATs). Similar to the gene signatures, changes in the levels of these transcripts are being studied for their use as diagnostic tool. In a first study, their expression levels accurately discriminate benign, localized tumor and metastatic prostate samples [142]. One of these transcripts, PCAT-1, seems to be a prostate-specific transcriptional repressor that regulates cell proliferation and that may hence have an important role in PCa progression [142]. Similar approaches might contribute to identify additional disease-associated lncRNAs that may further improve the stratification of cancer subtypes.

## 7. A Role of AR in PCa

**Figure 2:**
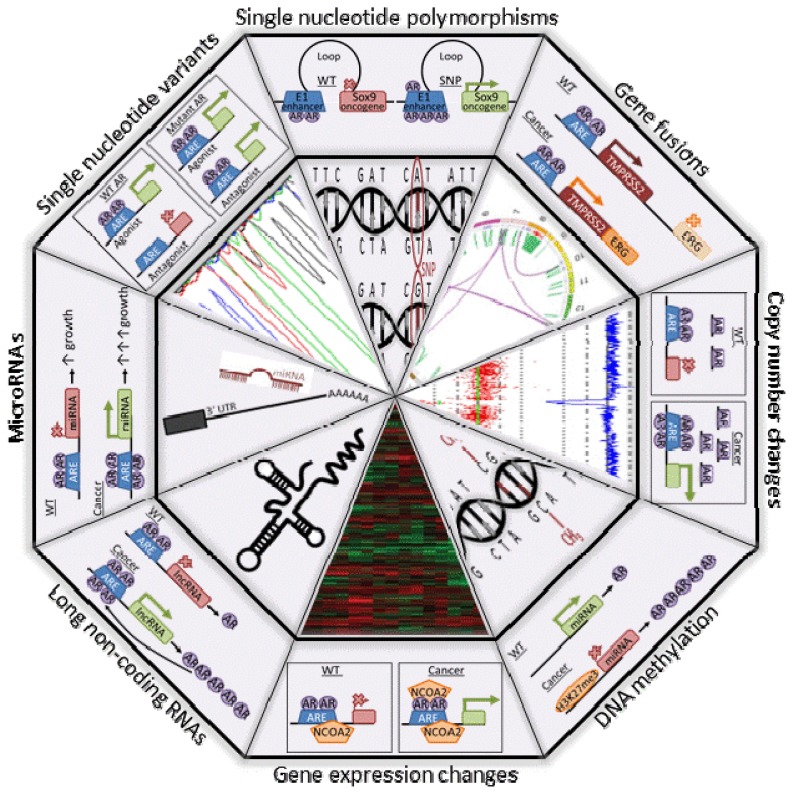
The genomic landscape of PCa. The integrated analysis of DNA, RNA and methylation data obtained with next-generation sequencing will help elucidate all relevant (epi)genetic changes in cancers (inner). Involvement of the androgen receptor in PCa tumorigenesis and progression can be demonstrated at all levels (outer).

Throughout this review, it became clear that the AR protein is a crucial transcription factor in normal, as well as diseased prostate and that it plays a pivotal role both during development and progression of PCa. As a transcription factor, it controls proliferation, as well as differentiation of prostate cells by regulating processes at multiple levels (proteins, miRNAs and lncRNAs) [143]. The AR gene itself is a target for (de-)methylation, amplification and mutagenesis that lead to gain of function. Many of the events described above not only affect the AR gene itself, but the entire pathway, for example, by disturbing AR cofactors. Since its dramatic effects on chromatin structure, the AR is now also a prime suspect to play a role in many genomic events, like translocations. It is even becoming more and more clear that part of the genetic predisposition to PCa also involves the AR at some level, for example, through the effect of SNPs on androgen response elements. All these processes are illustrated in Figure 2.

## 8. Conclusions

Carcinogenesis is a complicated integration of alterations of multiple transduction pathways as a result of changes at different levels, including the genome, epigenome, transcriptome, proteome, metabolome and lipidome. From the studies summarized in this review, it is clear that a major challenge is the identification of the driving events that could become therapeutic targets. This identification will only be possible by the study of larger cohorts of patients, as well as by a better understanding of the functional consequences of these PCa associated alterations. We would hope that the smart integration of data from different system biology analyses will add an additional layer of knowledge. This is exemplified in the study of Sharma *et al*., where cancer-specific changes in DNA binding by the AR, as well as other transcription factors were identified [144]. Indeed, while the first genomic analyses focused on the cancer exomes, it is likely that changes in the cistromes or transcription factor binding sites will play an oncogenic role.

In PCa, as in many other cancers, there is a considerable amount of interindividual tumor heterogeneity, both at the genetic and epigenetic level. This heterogeneity challenges the “one-size-fits-all” approaches for cancer management and highlights the need for individualized treatment approaches. In conclusion, the introduction of next-generation sequencing in the clinic is an important step forward, even when much more work is still required to fully understand the oncogenome and to integrate it with the other available system biology approaches.

## Figures and Tables

**Table 1:** Overview of v-ets erythroblastosis virus E26 oncogenes (ETS gene) fusions detected in samples of prostate cancer (PCa) patients. The list is organized according to the 3′ fusion partner.

5′ partner	3′ partner	Reference	5′ partner	3′ partner	Reference
TMPRSS2	ERG	[18]	TMPRSS2	ETV1	[18]
HERPUD1	ERG	[33]	SLC45A3	ETV1	[25]
SLC45A3	ERG	[29]	C15orf21	ETV1	[25]
NDRG1	ERG	[34]	HNRPA2B1	ETV1	[25]
FKBP5	ERG	[26]	FLJ35294	ETV1	[29]
TMPRSS2	ETV4	[19]	ACSL3	ETV1	[35]
DDX5	ETV4	[29]	EST14	ETV1	[36]
CANT1	ETV4	[37]	HERVK17	ETV1	[36]
KLK2	ETV4	[37]	HERVK22q11.23	ETV1	[25]
TMPRSS2	ETV5	[30]	FOXP1	ETV1	[36]
SLC45A3	ETV5	[30]	KLK2	ETV1	[26]
SLC45A3	FLI1	[38]	FUBP1	ETV1	[39]
SLC45A3	ELK4	[27]	SNURF	ETV1	[32]

**Table 2:** Single nucleotide polymorphisms (SNPs) associated with PCa-risk identified through genome-wide association studies (GWAS) across multiple cohorts.

Nearest Known Gene Within 100 kb	Chromosomal Locus	SNP	Region	References	OR a
KCNN3	1q23	rs1218582	Intronic	[75]	1.03–1.09
MDM4	1q32	rs4245739	Exonic/Coding	[75]	0.88–0.95
GGCX	2p11	rs10187424	Intergenic	[76]	1.06–1.19
EHBP1	2p15	rs721048	Intronic	[77]	1.15
THADA	2p21	rs1465618	Intronic	[78]	1.16–1.20
TAF1B:GRHL1	2p25	rs11902236	Intronic	[75]	1.03–1.10
ITGA6	2q31	rs12621278	Intronic	[78]	1.32–1.47
MLPH	2q37	rs2292884	Intronic	[79]	1.14
FARP2	2q37	rs3771570	Intronic	[75]	1.08–1.17
VGLL3	3p12	rs2660753	Intergenic	[80]	1.11–1.48
SIDT1	3q13	rs7611694	Intronic	[75]	0.88–0.93
EEFSEC	3q21	rs10934853	Intronic	[81]	1.12
ZBTB38	3q23	rs6763931	Intronic	[79]	1.04–1.18
CLDN11	3q26	rs10936632	Intergenic	[76]	1.08–1.28
AFM, RASSF6	4q13	rs1894292	Intronic	[75]	0.89–0.94
PDLIM5	4q22	rs12500426	Intronic	[78]	1.14–1.17
PDLIM5	4q22	rs17021918	Intronic	[78]	1.12–1.25
TET2	4q24	rs7679673	Intergenic	[78]	1.15–1.37
FGF10	5p12	rs2121875	Intronic	[76]	1.05–1.11
TERT	5p15	rs2242652	Intronic	[79]	1.15–1.39
FAM44B (BOD1)	5q35	rs6869841	Intergenic	[75]	1.04–1.11
CCHCR1	6p21	rs130067	Exonic/Coding	[79]	1.05–1.20
NOTCH4	6p21	rs3096702	Intergenic	[75]	1.04–1.10
ARMC2, SESN1	6q21	rs2273669	Intronic	[75]	1.03–1.11
SLC22A3	6q25	rs9364554	Intronic	[80]	1.17–1.26
RSG17	6q25	rs1933488	Intronic	[75]	0.87–0.92
JAZF1	7p15	rs10486567	Intronic	[82]	1.12–1.35
SP8	7p21	rs12155172	Intergenic	[75]	1.07–1.15
LMTK2	7q21	rs6465657	Intronic	[80]	1.03–1.19
SLC25A37	8p21	rs2928679	Intergenic	[78]	1.16–1.26
NKX3-1	8p21	rs1512268	Intergenic	[78]	1.13–1.28
EBF2	8p21	rs11135910	Intronic	[75]	1.07–1.16
None	8q24	rs10086908	Intergenic	[83]	1.14–1.25
None	8q24	rs7841060	Intergenic	[84]	1.19
None	8q24	rs13254738	Intergenic	[85]	1.11
None	8q24	rs16901979	Intergenic	[86]	1.66
None	8q24	rs16902094	Intergenic	[81]	1.21
None	8q24	rs445114	Intergenic	[81]	1.14
None	8q24	rs620861	Intergenic	[83,84]	1.11–1.28
None	8q24	rs6983267	Intergenic	[82,83,85,87]	1.13–1.42
None	8q24	rs7000448	Intergenic	[85]	1.14
None	8q24	rs1447295	Intergenic	[86–88]	1.29–1.72
MSMB	10q11	rs10993994	Intergenic	[80]	1.15–1.42
TRIM8	10q24	rs3850699	Intronic	[75]	0.89–0.94
CTBP2	10q26	rs4962416	Intronic	[82]	1.17–1.20
TH	11p15	rs7127900	Intergenic	[78]	1.29–1.40
MYEOV	11q13	rs11228565	Intergenic	[81]	1.23
MYEOV	11q13	rs7931342	Intergenic	[80]	1.19–1.25
MYEOV	11q13	rs10896449	Intergenic	[89]	1.09–1.20
MYEOV	11q13	rs12793759	Intergenic	[89]	1.04–1.18
MYEOV	11q13	rs10896438	Intergenic	[89]	1.02–1.12
MMP7	11q22	rs11568818	Intergenic	[75]	0.88–0.94
KRT8	12q13	rs902774	Intergenic	[79]	1.17
TUBA1C	12q13	rs10875943	Intergenic	[76]	1.02–1.18
TBX5	12q24	rs1270884	Intergenic	[75]	1.04–1.10
FERMT2	14q22	rs8008270	Intronic	[75]	0.86–0.93
RAD51L1	14q24	rs7141529	Intronic	[75]	1.06–1.12
VPS53, FAM57A	17p13	rs684232	Intergenic	[75]	1.07–1.14
HNF1B	17q12	rs11649743	Intronic	[90]	1.28
HNF1B	17q12	rs4430796	Intronic	[90,91]	1.16–1.38
HOXB13	17q21	rs11650494	Intergenic	[75]	1.09–1.22
None	17q24	rs1859962	Intergenic	[91]	1.20
SALL3	18q23	rs7241993	Intergenic	[75]	0.89–0.95
PPP1R14A	19q13	rs8102476	Intergenic	[81]	1.12
KLK3	19q13	rs2735839	Intergenic	[80]	1.25–1.72
GATAS, CABLES2	20q13	rs2427345	Intergenic	[75]	0.91–0.97
ZGPAT	20q13	rs6062509	Intronic	[75]	0.86–0.92
BIK	22q13	rs5759167	Intergenic	[78]	1.14–1.20
NUDT11	Xp11	rs5945619	Intergenic	[80]	1.19–1.46
SHROOM2	Xp22	rs2405942	Intronic	[75]	0.83–0.92
AR	Xq12	rs5919432	Intergenic	[79]	1.06–1.14

a OR, odds ratio, reported as a range across the various stages of GWAS discovery and validation when available.

## Abbreviations

AR: androgen receptor
CNA: copy number alteration
CRPC: castration-resistant prostate cancer
EMT: epithelial-to-mesenchymal transition
ETS: v-ets erythroblastosis virus E26 oncogene
FFPE: formalin fixed paraffin embedded
GWAS: genome-wide association study
indels: short insertions and deletions
lncRNA: long non-coding RNA
PCa: prostate cancer
PIN: prostatic intraepithelial neoplasia
PSA: prostate-specific antigen
SNP: single nucleotide polymorphism
SNV: single nucleotide variant
TMPRSS2: transmembrane protease serine 2
UTR: untranslated region
